# Portal Hypertensive Duodenal Polyps in a Pediatric Patient

**DOI:** 10.14309/crj.0000000000000476

**Published:** 2020-11-19

**Authors:** Shaik Naseerudin, Rishi Bolia

**Affiliations:** 1 Department of Pediatrics, All India Institute of Medical Sciences, Rishikesh, Uttarakhand, India

## CASE REPORT

**Figure 1.**
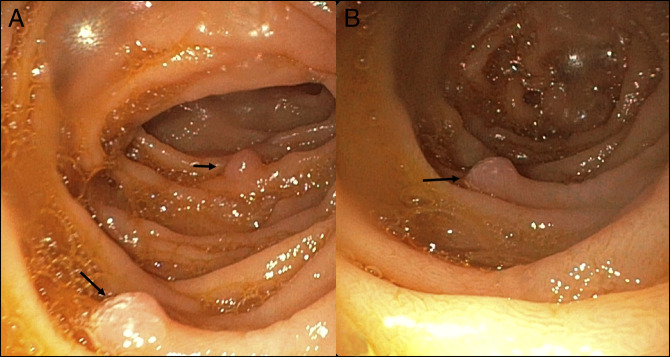
(A and B) Small sessile polyps in the second and third part of the duodenum.

**Figure 2.**
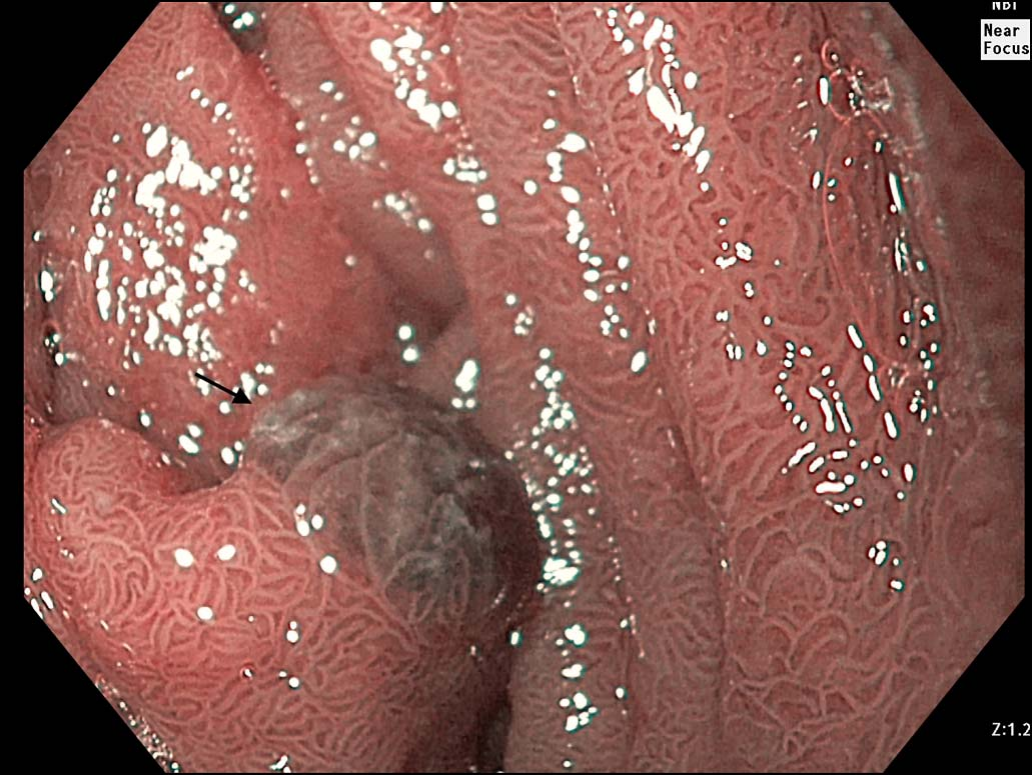
Narrow-band imaging suggesting increased vascularity of the polyp.

A 15-year-old adolescent girl presented with well-tolerated large volume hematemesis. She had no significant illnesses in the past. On examination, she had pallor and splenomegaly (∼7 cm below left costal margin). There was no jaundice. She underwent an ultrasound doppler that suggested extrahepatic portal vein obstruction. After stabilization, she underwent a gastroscopy, which showed large esophageal varices with a “white-nipple” sign indicative of recent bleeding. Endoscopic variceal ligation was performed. On further endoscopic evaluation, 3 small (∼3–5 mm) sessile polyps were seen in the second part of the duodenum (Figure 1). Narrow-band imaging rendered it black, suggesting increased vascularity (Figure 2). Snare polypectomy was performed. There was a small amount of self-limiting bleeding after the polypectomy. Histopathology showed numerous thick-walled capillaries in the subepithelial portion, suggesting a portal hypertensive polyp (PHP).

PHPs have been described in individuals with portal hypertension, adding on to the list of other gastrointestinal abnormalities such as gastropathy, antral vascular ectasia, enteropathy, colopathy, and others that have been described earlier. They have been reported in 1%–2.5% of adults undergoing upper gastrointestinal endoscopy for portal hypertension.^1^ PHPs have been mainly described in the stomach and less commonly elsewhere in the gastrointestinal tract. Duodenal involvement is uncommon, with a prevalence of 0.2%–0.8% in cirrhotic adults.^1,2^ To the best of our knowledge, there is only 1 previous report of PHPs in children.^3^ Devadson et al reported multiple duodenal PHPs (similar to our case) in 3 children with extrahepatic portal vein obstruction.

There are no diagnostic criteria for PHPs, but the presence of proliferating capillaries in the lamina propria indicates their portal hypertensive nature. The clinical significance of these polyps is unclear. They are mostly asymptomatic and have been suggested to be responsible for gastrointestinal bleeding, although there is insufficient data to definitely implicate them.^4,5^ It has been postulated that polyps develop because of neovascularization secondary to high portal pressure. Factors such as long-term proton pump inhibitor use, *Helicobacter pylori* infection, presence of multiple congenital anomalies, and endoscopic treatment for portal hypertension at an early age have been implicated in its pathogenesis, none of which were present in our child.^3^

It is not clear what happens during the follow-ups. In the report by Devadson et al, the findings persisted during the 6 years of follow-up.^3^ Whether they have a malignant potential is also unknown, but the fact that there is no increased risk of gastrointestinal cancers in patients with portal hypertension is reassuring and makes it unlikely. We believe that until we have more data about their relevance, simple watchful observation would suffice in such children. To conclude, we report an unusual gastrointestinal manifestation of portal hypertension in a child. Whether they are uncommon or have simply been underappreciated in children is a matter of speculation.

## DISCLOSURE

Author contributions: S. Naseerudin wrote the manuscript and approved the final manuscript. R. Bolia revised the manuscript for intellectual content, approved the final manuscript, and is the article guarantor.

Financial disclosure: None to report.

Informed consent was obtained for this case report.
